# Diagnostic and Prognostic Roles of C-Reactive Protein, Procalcitonin, and Presepsin in Acute Kidney Injury Patients Initiating Continuous Renal Replacement Therapy

**DOI:** 10.3390/diagnostics13040777

**Published:** 2023-02-18

**Authors:** Suyeon Han, Moo-Jun Kim, Ho-Joon Ko, Eu-Jin Lee, Hae-Ri Kim, Jae-Wan Jeon, Young-Rok Ham, Ki-Ryang Na, Kang-Wook Lee, Song-I. Lee, Dae-Eun Choi, Heyrim Park

**Affiliations:** 1 Department of Nephrology, Chungnam National University Hospital, Daejeon 35015, Republic of Korea; 2 Department of Nephrology, Chungnam National University Sejong Hospital, Sejong 30099, Republic of Korea; 3 Department of Pulmonary and Critical Care Medicine, Chungnam National University Hospital, Daejeon 35015, Republic of Korea; 4 Department of Medical Science, Medical School, Chungnam National University, Daejeon 35015, Republic of Korea

**Keywords:** CRP, procalcitonin, presepsin, CRRT, AKI, sepsis

## Abstract

For reducing the high mortality rate of severe acute kidney injury (AKI) patients initiating continuous renal replacement therapy (CRRT), diagnosing sepsis and predicting prognosis are essential. However, with reduced renal function, biomarkers for diagnosing sepsis and predicting prognosis are unclear. This study aimed to assess whether C-reactive protein (CRP), procalcitonin, and presepsin could be used to diagnose sepsis and predict mortality in patients with impaired renal function initiating CRRT. This was a single-center, retrospective study involving 127 patients who initiated CRRT. Patients were divided into sepsis and non-sepsis groups according to the SEPSIS-3 criteria. Of the 127 patients, 90 were in the sepsis group and 37 were in the non-sepsis group. Cox regression analysis was performed to determine the association between the biomarkers (CRP, procalcitonin, and presepsin) and survival. CRP and procalcitonin were superior to presepsin for diagnosing sepsis. Presepsin was closely related to the estimated glomerular filtration rate (eGFR) (*r* = −0.251, *p* = 0.004). These biomarkers were also evaluated as prognostic markers. Procalcitonin levels ≥3 ng/mL and CRP levels ≥31 mg/L were associated with higher all-cause mortality using Kaplan–Meier curve analysis. (log-rank test *p* = 0.017 and *p* = 0.014, respectively). In addition, procalcitonin levels ≥3 ng/mL and CRP levels ≥31 mg/L were associated with higher mortality in univariate Cox proportional hazards model analysis. In conclusion, a higher lactic acid, sequential organ failure assessment score, eGFR, and a lower albumin level have prognostic value to predict mortality in patients with sepsis initiating CRRT. Moreover, among these biomarkers, procalcitonin and CRP are significant factors for predicting the survival of AKI patients with sepsis-initiating CRRT.

## 1. Introduction

Continuous renal replacement therapy (CRRT) is a broadly used modality in the intensive care unit (ICU) to dialyze critically ill patients. Despite intensive care, acute kidney injury (AKI) patients undergoing CRRT have been reported to have high mortality rates of approximately 60–70% [1,2]. The identification of factors affecting the prognosis of patients with severe AKI undergoing CRRT is essential to improve patient survival. However, the prognostic factors in patients with severe AKI are affected by various factors, such as the accumulation of various uremic factors and electrolyte imbalance, as well as a decrease in material removal capacity owing to a decrease in kidney function [3,4].

C-reactive protein (CRP), procalcitonin, and presepsin have been suggested as representative diagnostic and prognostic factors in critically ill patients with sepsis. CRP is mainly produced in hepatocytes owing to the induction of inflammatory cytokines. CRP levels increase in both infection-induced and non-infectious inflammatory responses [5]. Procalcitonin is a 116-amino acid molecule with a size of 13 kD. It is an acid protein that is a precursor of the hormone calcitonin and is produced by thyroid C cells and neuroendocrine cells of the lungs and intestines [6]. Procalcitonin is present at low levels in normal people, but its levels increase greatly under various conditions (inflammation due to infection, tumor, burn, etc.; trauma; and surgery) [7]. Clinically, its levels greatly increase in bacterial and fungal infections but not in viral or non-infectious inflammation [8]. Presepsin is a cell surface glycoprotein expressed on macrophages and monocytes in response to pathogens. When monocytes are activated by an infectious microorganism, presepsin (soluble CD14 subtype) is released into the plasma, and its levels increase mainly in infectious inflammation [9].

It has been reported that the amount of presepsin in the blood increases with decreased renal function [10]. It has been known that CRP is also negatively correlated with the glomerular filtration rate (GFR), which is an indicator of renal function [11]. However, CRP can accurately predict infection in patients with impaired renal function [12]. Procalcitonin is affected by infection and sepsis, but it is also known to be affected by kidney function [13]. It has not been clearly identified whether CRP, procalcitonin, and presepsin could be prognostic and diagnostic markers in sepsis patients with impaired renal function undergoing CRRT.

This study aimed to assess whether CRP, procalcitonin, and presepsin could be used to diagnose sepsis and predict mortality in patients with impaired renal function undergoing CRRT.

## 2. Material and Methods

### 2.1. Study Design and Participants

This study was a single-center, retrospective, observational study at the Chungnam National University Hospital, Daejeon, Republic of Korea. We reviewed the medical records of patients who underwent CRRT in the ICU from 4 May 2019 to 1 October 2022 and underwent CRP, procalcitonin, and presepsin testing. Patients who underwent CRP, procalcitonin, and presepsin testing a day before CRRT or on the day of CRRT were included. Patients who did not undergo CRP, procalcitonin, and presepsin testing at CRRT initiation were excluded. Moreover, patients with end-stage renal disease who underwent regular dialysis were excluded.

We checked the white blood cell count, hemoglobin level, platelet count, creatinine level, estimated GFR (eGFR), albumin level, lactate level, Na level, P level, total Ca level, K level, and sequential organ failure assessment (SOFA) score on the day of CRRT initiation. If laboratory data were not available on the day of CRRT initiation, we considered data from a day around CRRT initiation. The SOFA score assesses the performance of body systems and consists of six categories (Glasgow Coma Scale, mean arterial pressure or administration of vasopressors, PaO_2_/FiO_2_ [mmHg (kPa)], platelet count [×10^3^/μL], bilirubin level [mg/dL], and creatinine level [mg/dL]). The SOFA score was assessed on the day of CRRT initiation.

Participants were divided into sepsis and non-sepsis groups according to the SEPSIS-3 definition on the day of CRRT initiation [14]. Mortality was defined as death after CRRT initiation, and 30-day mortality was defined as death within 30 days after CRRT initiation. The protocol for this study was reviewed and approved by the Institutional Review Boards of Chungnam National University Hospital (No. 2023-01-026).

### 2.2. Measurement

Renal function was assessed according to the eGFR based on creatinine, using the Modification of Diet in Renal Disease (MDRD) equation. Serum CRP levels were measured using an immunoturbidimetric assay (CRPL3; Roche Diagnostics, Indianapolis, IN, USA), and the reference range was 0–0.5 mg/dL. Plasma presepsin levels were measured using an automated chemiluminescent enzyme immunoassay (PATHFAST system; LSI Medience Corporation, Tokyo, Japan), and the reference limit was 300 pg/mL. Procalcitonin levels were measured using the Elecsys BRAHMS procalcitonin automated electrochemiluminescence assay (BRAHMS, Henningsdorf, Germany) on the Roche Cobas e-System (Roche Diagnostics, Basel, Switzerland), and the reference range was 0–0.05 ng/mL. Levels of total Ca, P, Na, K, albumin, and creatinine were analyzed using an automatic chemical analyzer (TBA-FX8; Toshiba, Tokyo, Japan).

### 2.3. Statistical Analysis

Clinical characteristics and outcomes were compared between the sepsis and non-sepsis groups. Continuous variables, which have been presented as mean and range (minimum-maximum), were compared using the Mann–Whitney test. Categorical variables were compared using the chi-square test or Fisher’s exact test. The diagnostic performance of CRP, procalcitonin, and presepsin to discriminate sepsis was analyzed using the area under the receiver operating characteristic (ROC) curve. The Optimal cutoff values of CRP, procalcitonin, and presepsin for predicting death were set as the maximum values of the specificity and sensitivity summation. Kaplan–Meier curve analysis and log-rank tests were performed according to the cutoff values of CRP, procalcitonin and presepsin. Univariate and multivariate Cox hazard model analyses were performed to evaluate the risk factors of death. All statistical analyses were performed using SPSS version 26.0 (IBM Corp., Armonk, NY, USA). A *p* value of <0.05 was considered statistically significant.

## 3. Results

### 3.1. Baseline Characteristics of the Study Population

**Figure 1:**
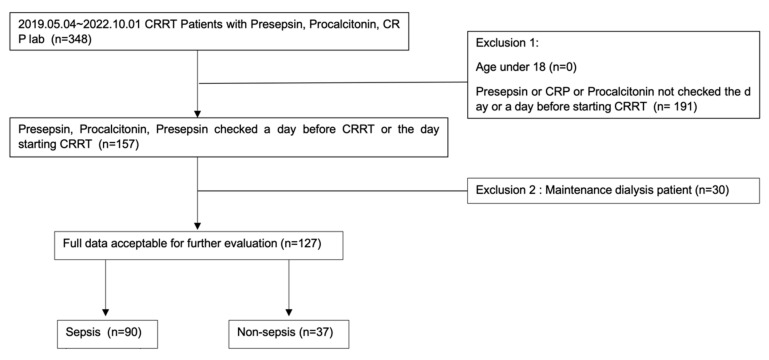
Study flowchart.

A total of 127 patients who had CRP, procalcitonin, and presepsin testing at the time of CRRT initiation were included. Of the 127 patients, 90 were included in the sepsis group and 37 were included in the non-sepsis group according to the SEPSIS-3 criteria [14] (Figure 1). Table 1 shows the baseline characteristics of patients in the sepsis and non-sepsis groups. Albumin was significantly lower in the sepsis group than in the non-sepsis group (*p* = 0.002). On the other hand, CRP, procalcitonin, and presepsin were significantly higher in the sepsis group than in the non-sepsis group (*p* < 0.004). Among 90 patients in the sepsis group, 29 (32%) had blood culture growth. Table 2 shows the principal diagnoses (cardiovascular, hepatobiliary, renal, respiratory, and bleeding disorders) in the 37 patients from the non-sepsis group.

### 3.2. Diagnostic Value of CRP, Procalcitonin, and Presepsin

**Figure 2:**
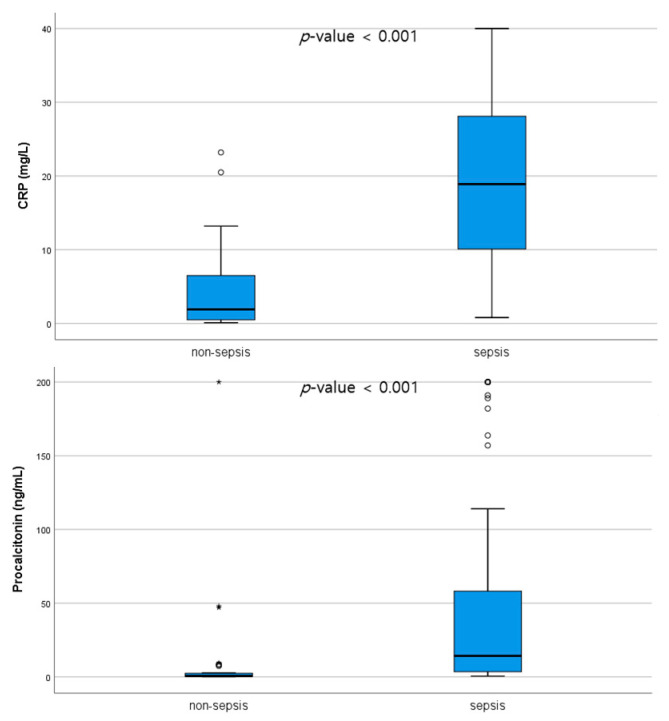

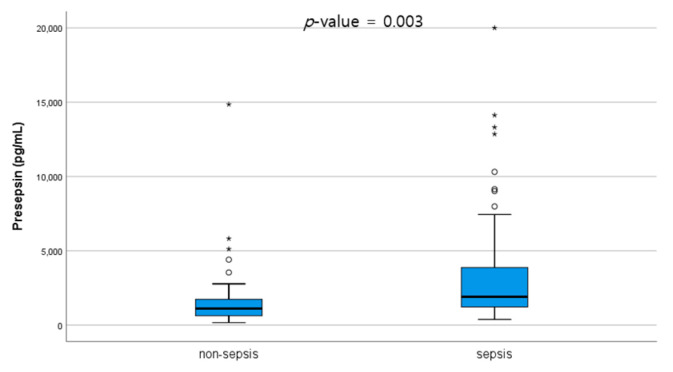
CRP, procalcitonin, and presepsin levels in the non-sepsis and sepsis groups. ° outlier, * extreme outlier.

**Figure 3:**
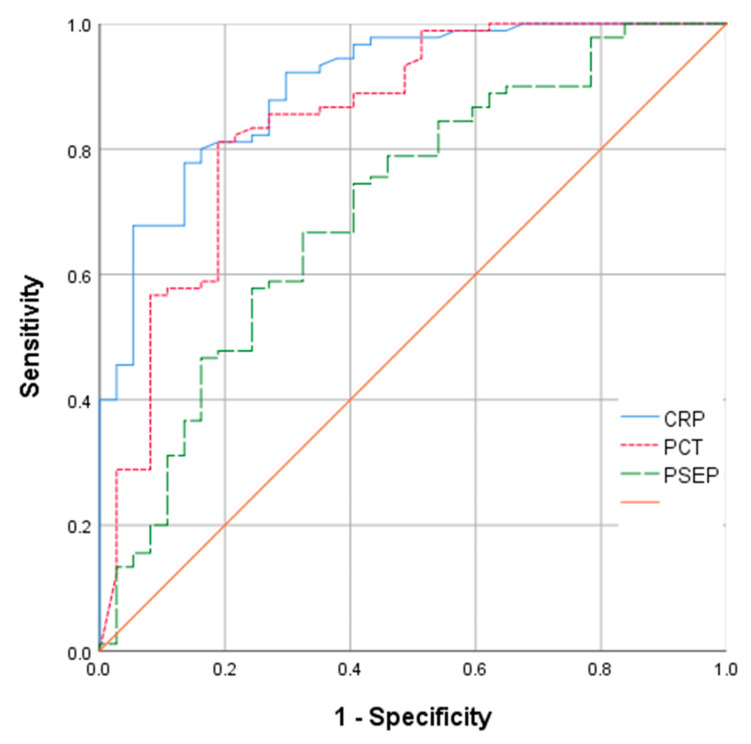
ROC analysis comparing the sensitivity and specificity of CRP, procalcitonin, and presepsin for discriminating sepsis.

Comparisons of CRP, procalcitonin, and presepsin levels between the sepsis and non-sepsis groups are shown in Figure 2. CRP, procalcitonin, and presepsin levels were higher in the sepsis group than in the non-sepsis group (Figure 2). ROC curve analysis results of CRP, procalcitonin, and presepsin to discriminate between the sepsis and non-sepsis groups are shown in Figure 3 and Table 3. According to this analysis, the AUC of CRP was 0.901 (95%CI: 0.844–0.958, *p* < 0.001), and the cutoff value was 9.05 mg/L. The AUC of procalcitonin was 0.845 (95%CI: 0.765–0.958, *p* < 0.001), and the cutoff value was 2.86 ng/mL. The AUC of presepsin was 0.706 (95%CI: 0.604–808, *p* < 0.001), and the cutoff value was 1390 pg/mL. According to our study, in patients initiating CRRT, CRP, and procalcitonin were more sensitive than presepsin for sepsis diagnosis.

### 3.3. Correlation between CRP, Procalcitonin, and Presepsin with Creatinine and eGFR 

Presepsin was correlated with creatinine (rho = 0.180, *p* = 0.043) and eGFR (rho = −0.251, *p* = 0.004). However, procalcitonin was not correlated with creatinine (rho = −0.014, *p* = 0.873) or eGFR (rho = −0.137, *p* = 0.124). Moreover, CRP was not correlated with creatinine (rho = −0.036, *p* = 0.689) or eGFR (rho = −0.079, *p* = 0.377).

### 3.4. Prognostic Value of Procalcitonin and CRP in Sepsis

The baseline characteristics of the 30-day survivors and the 30-day non-survivors are summarized in Supplementary Table S1. The albumin level and platelet count were significantly higher, and the SOFA score and lactate level were significantly lower among the 30-day survivors than among the 30-day non-survivors.

We analyzed whether CRP, procalcitonin, and presepsin were associated with survival in the sepsis group. Appropriate cutoffs to predict death were obtained (Supplementary Table S2). Procalcitonin and CRP were found to be independent factors for predicting survival with certain cutoff values. However, presepsin was not associated with survival.

**Figure 4:**
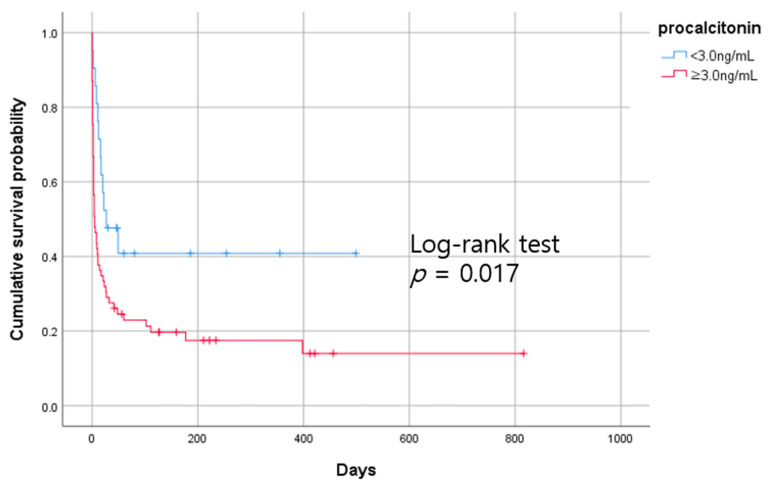
Kaplan–Meier analysis of all-cause mortality for procalcitonin in patients with sepsis who initiated CRRT, and log-rank test.

The optimal cutoff value of procalcitonin for predicting death was 3.00 ng/mL in patients with sepsis, which was similar to the 1st quartile value of procalcitonin (3.39 ng/mL). Kaplan—Meier survival curve analysis showed that mortality was higher in patients with high procalcitonin levels (≥3 ng/mL) than in those with low procalcitonin levels (<3 ng/mL) (log-rank test *p* = 0.017) (Figure 4).

**Figure 5:**
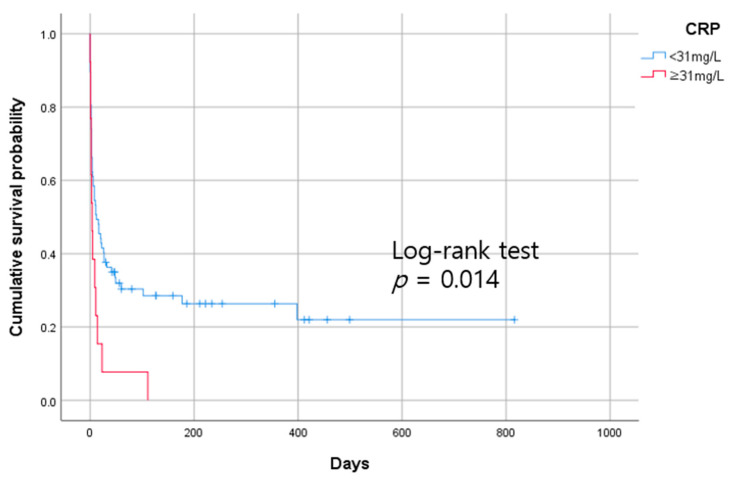
Kaplan–Meier analysis of all-cause mortality for CRP in patients with sepsis who initiated CRRT, and log-rank test.

The optimal cutoff value of CRP for predicting death was 31 mg/L in patients with sepsis, which was between the 3rd and 4th quartile values of CRP (28.2 mg/L and 40 mg/L, respectively). Kaplan–Meier survival curve analysis showed that mortality was higher in patients with high CRP levels (≥31 mg/L) than in those with low CRP levels (<31 mg/L) (log-rank test *p* = 0.014) (Figure 5).

The optimal cutoff value of presepsin for predicting death was 693 pg/mL, and Kaplan–Meier survival curve analysis was performed, but the log-rank test *p*-value was not significant (*p* = 0.144) (Supplementary Figure S1).

Procalcitonin was negatively correlated with the platelet count (rho = −0.220, *p* = 0.013), albumin level (rho = −0.185, *p* = 0.038), and total Ca level (rho = −0.231, *p* = 0.009), and was positively correlated with the CRP level (rho = 0.471, *p* < 0.001) and SOFA score (rho = 0.175, *p* = 0.049) (Table 4). CRP and procalcitonin showed a strong significant positive correlation (*p* < 0.001).

Univariate and multivariate Cox proportional hazards model analyses of all-cause mortality are shown in Table 5. In unadjusted Cox proportional hazards analysis, platelet count, lactate level, albumin level, eGFR, SOFA score, phosphate level, procalcitonin level ≥3.00 ng/mL, and CRP level ≥31 mg/L were significantly associated with an increased risk of all-cause mortality. After multivariate adjustment, CRP level ≥31 mg/L was a risk factor of death on adjusting age, platelet count, lactate level, albumin level, eGFR, and phosphate level (Table 5). Comorbidities were included in the univariate Cox proportional hazards model analyses (Supplementary Table S3). There was no significant relation between all-cause mortality and underlying diseases in patients with sepsis who initiated CRRT.

## 4. Discussion

We found that CRP, procalcitonin, and presepsin can discriminate sepsis with statistical significance. CRP (AUC 0.901) and procalcitonin (AUC 0.845) showed greater accuracy than presepsin (AUC 0.706) for diagnosing sepsis.

Studies have shown that severe AKI patients have different biomarker accuracies and cutoff values compared with non-AKI patients, and renal function significantly influences biomarker accuracy [15,16]. Our study patients were all initiating CRRT and were classified as having stage 3 AKI according to the KDIGO AKI criteria [17]. Nakamura et al. reported that in severe AKI patients, procalcitonin is significantly more accurate than presepsin for diagnosing sepsis [18]. In the stage 3 AKI subgroup, the AUC value of presepsin was significantly lower than that in the non-AKI group [18]. This result is consistent with our study result showing presepsin with the lowest AUC among sepsis markers (CRP, procalcitonin and presepsin). In our study, presepsin had a strong correlation with eGFR. Since presepsin is filtered in the glomerulus or catabolized by proximal tubular cells, renal dysfunction can delay presepsin clearance [19]. Severe AKI may confound the sepsis diagnosing ability of presepsin. Notably, presepsin did not affect mortality in our study. Presepsin levels ≥693 pg/mL showed lower cumulative survival probability in the Kaplan–Meier curve without statistical significance. Several past studies have shown that presepsin levels are higher in non-survivors than in survivors among non-AKI patients [20,21]. Collectively, it can be suggested that a decrease in renal function has a significant effect on the change in presepsin associated with sepsis.

In our study, procalcitonin showed no correlation with eGFR, although we could not prove that renal dysfunction has no influence on serum procalcitonin levels. Despite renal dysfunction, procalcitonin was a meaningful marker to diagnose sepsis and predict the prognosis of septic AKI in patients initiating CRRT in our study. Previous studies have shown that renal function impairment may elevate serum procalcitonin levels, partially by decreasing procalcitonin elimination from the kidney [10]. Procalcitonin is considered a predictor of AKI in various settings [22,23,24]. Despite the influence of renal function, some studies have shown that even in severe AKI patients, procalcitonin has diagnostic value to discriminate against sepsis [15,18]. Kan et al. described that the procalcitonin predictive ability for AKI seems to be blunted by infection [10].

Jekarl et al. and Enguix-Armada et al. reported that higher procalcitonin is associated with adverse prognosis [25,26]. However, these studies did not evaluate when renal function worsened in acute renal injury. There is limited information on the use of CRP, procalcitonin, and presepsin to predict survival in critically ill patients with sepsis undergoing CRRT. In this study, we found that procalcitonin was an important factor for predicting survival in AKI patients with sepsis-initiating CRRT. Procalcitonin levels ≥3 ng/mL had an HR of 2.061 (95 CI: 1.103–3.850) over procalcitonin levels <3 ng/mL. In recent studies, platelet count, albumin level, and SOFA score were identified as mortality factors in patients undergoing CRRT [27,28,29]. There was a statistically meaningful correlation between procalcitonin and platelet count, albumin level, SOFA score, and CRP level. In our study, among patients initiating CRRT, a higher lactate level, higher SOFA score, higher eGFR, and lower albumin level were associated with a worse prognosis. 

CRP has diagnostic value to discriminate sepsis and prognostic value to predict mortality in patients initiating CRRT. Park et al. reported that CRP can accurately predict infection in patients with renal dysfunction [12]. However, in AKI patients with sepsis initiating CRRT, the prognostic value of CRP has rarely been noted. In our study, CRP levels ≥ 31 mg/L had an HR of 2.079 (95 CI: 1.124–3.845) over CRP levels <31 mg/L. Adjusting for age, platelet count, lactate level, albumin level, eGFR, and phosphate level in the multivariate model, CRP ≥ 31 mg/L remained a prognostic factor for predicting survival. 

We used the eGFR according to the MDRD equation as a factor that can reflect renal function. This equation is from a large population of CKD patients and is usually applied to CKD patients. In AKI patients, creatinine, and volume change rapidly and neither creatinine nor eGFR would provide an accurate estimate of the GFR. A kinetic eGFR equation can estimate GFR in AKI patients [30]. Cystatin-C-based equations, urinary or plasma clearance of exogenous filtration markers, and urinary clearance of creatinine are more accurate methods to measure the GFR [31,32]. Assessing the GFR in AKI patients is challenging, and the assessment of biomarkers of renal cellular injury, such as KIM-1, NGAL, and IL-18, can be a new strategy to detect AKI [33].

However, some studies have shown the possibility of using eGFR as a marker for AKI. The RIFLE criteria included the eGFR change from baseline to diagnose AKI [34]. Robert et al. compared creatinine clearance, eGFR, and creatinine to predict contrast-induced AKI [35]. Renal function was measured at baseline and within 48 h after percutaneous coronary intervention using three methods (creatinine clearance using the Cockcroft–Gault method, eGFR using the MDRD equation, and serum creatinine) [35]. Creatinine clearance, eGFR, and serum creatinine can predict contrast-induced AKI well [35]. Kirwan et al. compared 4 h creatinine clearance with creatinine and cystatin-C-based eGFR equations in critically ill patients with AKI [36]. eGFR equations were not sufficiently accurate for use in critically ill patients with AKI [36]. However, eGFR equations were better than creatinine alone at predicting the 4 h creatinine clearance [36].

Despite all these limitations, we used creatinine-based eGFR (MDRD) to estimate kidney function in AKI patients because our study was a retrospective observational study with medical data, and we considered creatinine and eGFR based on creatinine. eGFR (MDRD) accounts for sex and age with creatinine levels; thus, it was used despite the above-mentioned limitations. Further studies should be conducted with more accurate GFR measures in AKI patients.

In our study, a higher eGFR was associated with higher mortality. In many studies on CKD patients, a lower eGFR was associated with higher mortality [37,38]. However, the relationship between mortality and eGFR in severe AKI patients has rarely been studied. In the MDRD equation, a lower creatinine level is associated with a higher eGFR. In critically ill patients, malnutrition, rapid muscle loss, and volume overload were associated with lower creatinine levels and higher mortality. Medina-Liabres et al. reported that lower serum creatinine levels were independently associated with an increased risk of death in patients initiating CRRT [39]. Haas et al. illustrated a U-shaped relationship between eGFR and all-cause mortality [40]. A high eGFR was associated with high mortality in these data [40]. The reasons for this could be early diabetic nephropathy, diet, medication use, rapidly changing kidney function, and abnormal muscle mass [40].

In our study, the baseline data for inflammatory biomarkers were collected on the day of initiating CRRT. Many studies collect baseline data on the day of admission to the ICU and additional laboratory data after ICU admission. However, at the time of admission, the patient population includes both patients with good kidney function and those with poor kidney function. We considered that this point in time could be a confounding factor to evaluate inflammatory biomarkers. Therefore, we obtained baseline data just before initiating CRRT, and we thought that renal function would be the lowest at this point.

Several limitations of our studies should be additionally considered. First, this study had a relatively small sample size and was a retrospective study at a single medical center. However, our study included over 100 renal failure patients and compared to the sample size in other studies, this number is not small. Second, the study included patients who underwent laboratory tests at the time of initiating CRRT (on the day of CRRT or the day before CRRT) and patients who did not undergo laboratory tests were excluded. This might have resulted in selection bias. Third, in our study, the baseline data were collected on the day of CRRT initiation and were collected only once. Serial changes of data were not considered in this study. Fourth, this was an observational study and could not demonstrate the causal risk association between factors (albumin level, lactate level, SOFA score, etc.) and death.

## 5. Conclusions

Among patients initiating CRRT, CRP, procalcitonin, and presepsin have diagnostic value to discriminate sepsis, and procalcitonin and CRP have higher accuracy to diagnose sepsis. A higher lactic acid level, SOFA score, and eGFR, and a lower albumin level have prognostic value to predict mortality. Prognosis is worse in patients with procalcitonin levels ≥ 3 ng/mL than in those with levels < 3 ng/mL. Moreover, mortality is higher in patients with CRP levels ≥ 31 mg/L than in those with CRP levels < 31 mg/L. Further prospective studies in large populations with serial data followup and more accurate kidney function markers are required to clarify the values of inflammatory biomarkers (CRP, procalcitonin, and presepsin) in severe AKI patients.

## Figures and Tables

**Table 1:** Baseline characteristics of the study population.

Variables	Total (*n* = 127)	Sepsis (*n* = 90)	Non-Sepsis (*n* = 37)	*p*-Value
Age, y	69 (29–97)	70.56 (39–97)	65 (29–89)	0.189
Male, n (%)	88 (69.3%)	64 (71.1%)	24 (64.9%)	0.679
Death, n (%)	95 (74.8%)	69 (76.7%)	26 (70.3%)	0.923
Site of Infection				
Respiratory		39 (43%)		
Genitourinary		8 (9%)		
Gastrointestinal		8 (9%)		
Soft tissue		6 (6%)		
Fungal		7 (7%)		
Others		22 (24%)		
Positive blood culture		29		
30-day mortality	81 (63.8%)	62 (68.9%)	19 (51.4%)	0.565
Hb (g/dL)	9.75 (3.8–18.80)	9.49 (3.8–15.10)	10.72 (4.90–18.80)	0.123
Albumin (g/dL)	2.74 (1.7–4.8)	2.63 (1.7–4.10)	3.03 (1.80–4.80)	0.002
Cr (mg/dL)	2.76 (0.43–22)	2.77 (0.43–22)	2.57 (0.83–6.93)	0.682
eGFR (mL/min/1.73 m^2^)	33.14 (2.0–145)	31.99 (2–93)	33.15 (7.0–74)	0.891
Phosphate (mg/dL)	5.12 (1.4–16)	5.20 (1.4–16)	4.91 (1.50–9.50)	0.613
Potassium (mEq/L)	4.47 (2.20–9.0)	4.48 (2.2–9)	4.26 (2.90–6.26)	0.560
Total Ca (mg/dL)	7.42 (5.50–9.70)	7.32 (5.50–9.40)	7.72 (5.90–9.70)	0.158
WBC (×10^9^/L)	12,910 (10–55,770)	12,614 (10–55,770)	13,002 (1020–29,400)	0.864
Platelet (×10^3^/µL)	120 (5–471)	110 (5–471)	147 (41–289)	0.186
CRP (mg/dL)	15.06 (0.1–40)	19.48 (0.8–40)	5 (0.50–23.20)	<0.001
Presepsin (pg/mL)	2802 (172–20,000)	3204 (386–20,000)	1578.95 (172–5822)	0.003
Procalcitonin (ng/mL)	38.97 (0.05–200)	51.04 (0.57–200)	5.78 (0.05–48)	<0.001
SOFA score	11.66 (4.0–19.0)	12.1 (4.0–19.0)	1578 (172–5822)	0.212
Lactate (mmol/L)	5.52 (0.70–20)	5.56 (0.70–20)	6.31 (1–17)	0.509

Hb hemoglobin, Cr creatinine, eGFR estimated glomerular filtration rate, WBC white blood cell.

**Table 2:** Principal diagnoses of non-sepsis CRRT patients (*n* = 37).

Organs	Main Principal Diagnoses	*n*
Cardiovascular (*n* = 13)	Heart failure	3
	MI	3
	PTE	2
	Pulmonary hypertension	1
	Aortic dissection	1
	Others	3
Respiratory (*n* = 1)	ILD	1
Hepatobiliary (*n* = 9)	Liver cirrhosis aggravation	3
	Hepatitis	5
	Others	1
Renal (*n* = 6)	CKD aggravation	3
	AKI	2
	Others	1
Bleeding (*n* = 3)	GI bleeding	1
	Spleen rupture	1
	Hematoma	1
Others (*n* = 5)	HLH	1
	Hanging	2
	Catecholamine crisis	1
	Vasculitis	1

**Table 3:** Diagnostic value of CRP, procalcitonin, and presepsin for sepsis.

Marker	AUC (95%CI)	*p* Value	Cut-Off Value	Sensitivity (%)	Specificity (%)
Presepsin	0.706 (0.604–0.808)	<0.001	1390 (pg/mL)	66.7	67.5
Procalcitonin	0.845 (0.765–0.958)	<0.001	2.86 (ng/mL)	81.1	81.0
CRP	0.901 (0.844–0.958)	<0.001	9.05 (mg/L)	77.8	86.4

**Table 4:** Pearson correlation coefficients for the relation of procalcitonin and other factors.

	Pearson Coefficient (r)	Significance (*p*-Value)
eGFR	−0.137	0.124
Presepsin	0.098	0.271
CRP	0.471	<0.001
Cr	−0.14	0.873
Age	−0.012	0.896
WBC	−0.044	0.621
Platelet	−0.220	0.013
Lactate	0.097	0.279
Albumin	−0.185	0.038
Hb	0.127	0.155
Na	−0.152	0.088
*p*	0.082	0.362
TCa	−0.231	0.009
K	−0.009	0.918
SOFA score	0.175	0.049

TCa: total calcium.

**Table 5:** Risk factors associated with all-cause mortality using Cox proportional hazards analysis.

	Univariate HR (95% CI)	*p*-Value	Multivariate HR (95% CI)	*p*-Value
Age	1.010 (0.990–1.031)	0.343	1.029 (1.006–1.052)	0.012
Sex	1.328 (0.773–2.280)	0.304		
Platelet	0.997 (0.994–1.000)	0.021	0.997 (0.995–1.000)	0.040
Lactate	1.136 (1.076–1.199)	<0.001	1.081 (1.015–1.151)	0.015
Albumin	0.443 (0.252–0.777)	0.005	0.487 (0.258–0.919)	0.026
Hb	0.922 (0.805–1.055)	0.239		
eGFR	1.015 (1.003–1.026)	0.010	1.014 (1.002–1.026)	0.019
Cr	0.941 (0.832–1.063)	0.326		
SOFA score	1.135 (1.061–1.213)	<0.001		
PCT ≥ 3.00 ng/mL	2.061 (1.103–3.850)	0.023		
CRP ≥ 31 mg/L	2.079 (1.124–3.845)	0.020	2.007 (1.036–3.887)	0.039
Presepsin ≥ 693 pg/mL	1.921 (0.771–4.782)	0.161		
K	1.062 (0.886–1.272)	0.517		
TCa	0.807 (0.610–1.067)	0.132		
*p*	1.105 (1.009–1.210)	0.032	1.165 (1.045–1.300)	0.039
Na	1.012 (0.979–1.046)	0.490		
CRP	1.010 (0.988–1.032)	0.395		
Presepsin	1.00 (1.00–1.00)	0.471		
PCT	1.002 (0.998–1.005)	0.306		

Hb hemoglobin, PCT procalcitonin, TCa total calcium.

## Data Availability

The datasets used and analyzed in the current study are available from the corresponding author upon reasonable request.

## Supplementary Materials

The following supporting information can be downloaded at: https://www.mdpi.com/article/10.3390/diagnostics13040777/s1. Supplementary Table S1. Baseline characteristics of 30-day survivors and 30-day non-survivors; Supplementary Table S2. Diagnostic tests of inflammatory markers for mortality; Supplementary Figure S1. Kaplan–Meier analysis of all-cause mortality for presepsin in patients with sepsis who initiated CRRT, and log-rank test; Supplementary Table S3. Underlying diseases associated with all-cause mortality by Cox proportional hazards analysis.
