# Supplementary material for: Diagnostic and Prognostic Roles of C-Reactive Protein, Procalcitonin, and Presepsin in Acute Kidney Injury Patients Initiating Continuous Renal Replacement Therapy
Source: Diagnostics (Basel). 2023 Feb 18;13(4):777. doi: 10.3390/diagnostics13040777 (PMC9955569; doi:10.3390/diagnostics13040777)
Supplement: Supplementary file 1 [file diagnostics-13-00777-s001.zip › diagnostics-2129829-supplementary.pdf]

	Total septic patients (n=90)	30day-Survivor (n=28)	30day-non-survivor (n=62)	p-value
Age, y	70.56 (39-97)	67.85 (39-97)	71.79 (42-93)	0.105
Male, n(%)	64 (71.1%)	18 (64.3%)	46 (74.2%)	0.337
Positive blood culture	39 (43.3%)	11 (39.3%)	28 (45.2%)	0.603
DM	31 (34.4%)	10 (35.7%)	21 (33.9%)	0.865
HTN	39 (43.3%)	10 (35.7%)	29 (46.8%)	0.327
HF	12 (13.3%)	5 (17.9%)	7 (11.3%)	0.396
IHD	6 (6.7%)	1 (3.6%)	5 (8.1%)	0.429
AF	14 (15.6%)	3 (10.7%)	11 (17.7%)	0.394
CI	22 (24.4%)	8 (28.6%)	14 (22.6%)	0.540
COPD	4 (4.4%)	1 (3.6%)	3 (4.8%)	0.787
LC	7 (7.8%)	0 (0%)	7 (11.3%)	0.064
CKD	24 (26.7%)	9 (32.1%)	15 (24.2%)	0.430
Malignancy	27 (30%)	9 (32.1%)	18 (29.0%)	0.766
Hb (g/dL)	9.49 (3.80-15.10)	9.79 (6.60-13.40)	9.36 (3.8-15.10)	0.236
Albumin (g/dL)	2.63 (1.7-4.10)	2.81 (2.0-4.10)	2.55 (1.7-3.5)	0.031
Cr (mg/dL)	2.77 (0.43-22)	3.16 (0.82-10.90)	2.60 (0.43-22)	0.063
eGFR (mL/min/1.73m^2)	31.99 (2.00-93)	24.88 (6.0 -68)	35.20 (2-93)	0.055
Phosphate(mg/d)	5.20 (1.40-16)	4.82 (1.6-12.6)	5.37 (1.4-16)	0.280
Potassium(mEq/L)	4.48 (2.2-9.0)	4.48 (2.64-8.30)	4.48 (2.20-9.00)	0.610
Total Ca(mg/dL)	7.32 (5.50.-9.40)	7.48 (5.5-8.6)	7.25 (5.50-9.40)	0.115
WBC (x10^9/L)	12614 (10-55770)	14792 (300-43230)	11630 (10-55770)	0.376
PLT (x10^3/uL)	110 (5-471)	151.92 (5-395)	92.38 (9-471)	0.008
CRP (mg/dL)	19.48 (0.8-40)	16.67 (1.2-38)	20.75 (0.80-40)	0.099
Presepsin (pg/mL)	3204.86 (386-20000)	3733.96 (386-14124)	2965.91 (389-20000)	0.480
Procalcitonin (ng/mL)	51.04 (0.57-200)	42.17 (0.57-200)	55.05 (0.62-200)	0.145
SOFA score	12.1 (4-19)	10.21 (4-19)	12.95 (7-19)	0.001
Lactate (mmol/L)	5.56 (0.70-20)	3.84 (0.70-11)	6.34 (1.3–20)	0.002

Supplement Table S1. Baseline characteristics of 30day-survivor and 30day-non-survivor

marker	Cut-off value	Sensitivity (%)	Specificity (%)
Presepsin	693 (pg/mL)	0.928	0.190
Procalcitonin	3.00 (ng/mL)	0.826	0.429
CRP	31 (mg/dL)	0.188	1.000

Supplement Table S2. Diagnostic test of inflammatory marker for death

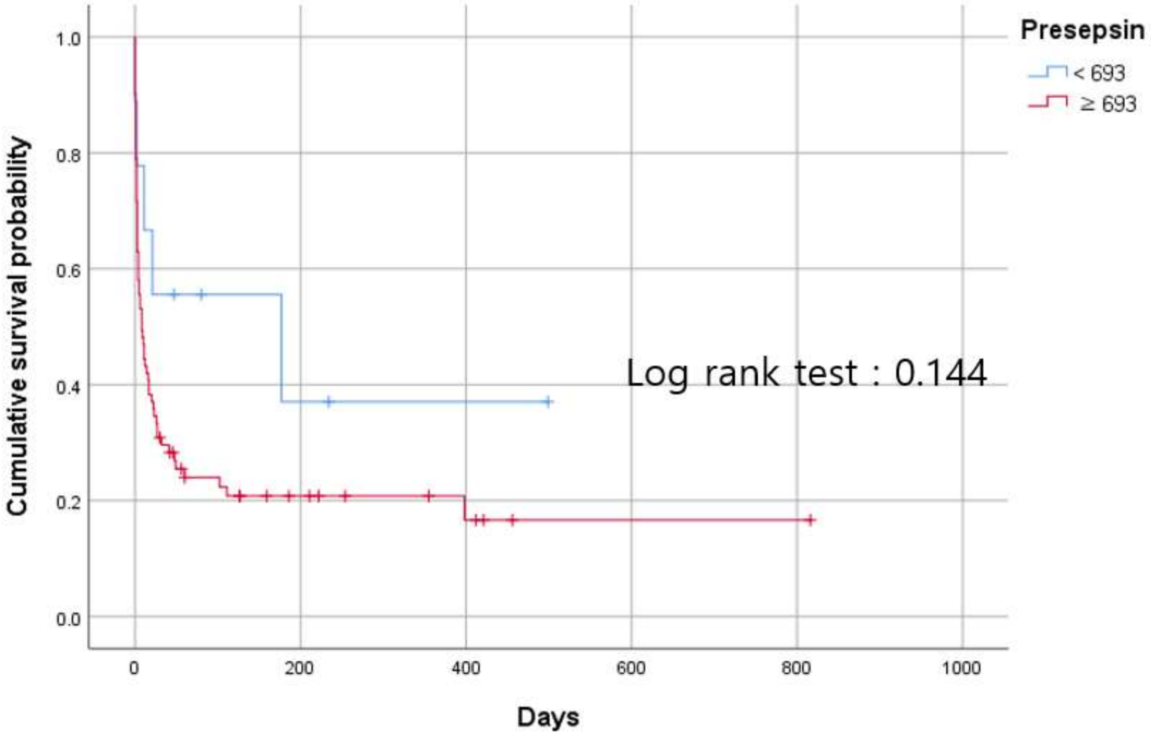

Supplement Figure S1. Kaplan-Meier analysis of all-cause mortality of presepsin in septic CRRT patients and log-rank test.

	Univariate HR (95% CI)	P value
Diabetes mellitus	0.826 (0.502-1.358)	0.450
Hypertension	0.994 (0.616-1.603)	0.980
Heart failure	0.687 (0.328-1.438)	0.319
Ischemic heart disease	1.251 (0.540-2.897)	0.601
Atrial fibrillation	0.987 (0.516-1.888)	0.969
Cerebral Ischemia	0.658 (0.371-1.167)	0.152
COPD	1.252 (0.392-3.997)	0.704
Liver cirrhosis	1.881 (0.854-4.144)	0.117
Chronic kidney disease	0.681(0.393-1.180)	0.170
Malignancy	1.089 (0.654-1.811)	0.744

COPD; Chronic obstructive pulmonary disease

Supplement Table S3. Underlying disease associated with all-cause mortality by Cox proportional hazard analysis.
